# Unmasking a Silent Persistent Hyaloid Artery: A Rare Cause of Vitreous Hemorrhage in an Elderly Patient

**DOI:** 10.7759/cureus.98980

**Published:** 2025-12-11

**Authors:** Eirini Maliagkani, Aikaterini Chatzara, Konstantina Chronopoulou, Ioannis Tservakis

**Affiliations:** 1 1st Department of Ophthalmology, National and Kapodistrian University of Athens Medical School, General Hospital of Athens "G. Gennimatas", Athens, GRC; 2 Ophthalmology Department, Eye Day Clinic, Athens, GRC

**Keywords:** intraoperative video, pars plana vitrectomy, persistent fetal vasculature, persistent hyaloid artery, vitreous hemorrhage

## Abstract

Persistent hyaloid artery (PHA) rupture is a rare cause of vitreous hemorrhage, particularly in older adults, as hyaloid remnants typically regress during early life. We report the case of a 70-year-old man who presented with sudden, painless vision loss in the left eye due to dense vitreous hemorrhage. Immediate pars plana vitrectomy was performed, during which an actively bleeding PHA was identified and successfully cauterized. Intraoperative video documentation provided direct visualization of the ruptured embryonic vessel, confirming the diagnosis. No retinal tears, retinal detachment, or neovascularization were noted. Postoperatively, the patient experienced progressive visual recovery, ultimately returning to his baseline visual acuity. This case expands the known age spectrum of symptomatic PHA, demonstrates the diagnostic and therapeutic value of early vitrectomy in non-clearing hemorrhage, and underscores the importance of considering congenital vascular remnants in the differential diagnosis of spontaneous vitreous hemorrhage in elderly patients.

## Introduction

The hyaloid artery, a branch of the ophthalmic artery, is an important component of the embryonic vasculature. Its branches form the vasa hyaloidea propria that nourishes the primary vitreous [1]. The hyaloid artery normally regresses by the early postnatal period; however, persistent remnants are found in approximately 3% of full-term newborns [2], and timely regression is crucial for transparent optical media development [3]. As the hyaloid vascular system becomes atrophic, a clear S-shaped channel, known as Cloquet’s canal, becomes visible within the developing vitreous cavity, extending from the space over the optic disc (area of Martegiani) to Berger’s retrolental space [4,5]. Incomplete involution may result in residual embryonic structures, such as Bergmeister’s papilla posteriorly or Mittendorf’s dot anteriorly, both typically associated with normal vision [1]. However, when extensive remnants are observed, they may be linked to cataract, amblyopia, strabismus, and nystagmus [6].

Any defect occurring along the normal involution pathway produces a distinct clinical phenotype. The spectrum of these congenital anomalies is collectively encompassed by the umbrella term "persistent fetal vasculature" (PFV). Unilateral involvement of PFV occurs in 95% of cases. Although rare, bilateral PFV has been reported, typically in conjunction with specific congenital syndromes [3].

Vitreous hemorrhage secondary to rupture of a persistent hyaloid artery (PHA) is exceedingly rare, typically occurring spontaneously or following trauma, and in some cases, associated with rapid eye movement (REM) sleep or elevated blood pressure [1,7].

We present a case of spontaneous rupture of a posterior PHA remnant in a 70-year-old male, resulting in dense vitreous hemorrhage. We discuss the diagnostic dilemma and highlight the role of early pars plana vitrectomy and endocautery of the embryonic vessel, which led to excellent anatomical and visual outcomes.

## Case presentation

A 70-year-old man presented with sudden, painless loss of vision in his left eye. One month earlier, he noticed floaters in the same eye, for which fundoscopy revealed no retinal pathology. No optical coherence tomography (OCT) or fundus photography was performed at that visit; only dilated fundoscopy was available. At the presentation, he reported an abrupt onset of blurred vision, followed within minutes by profound visual loss. Fundus examination was obscured by dense vitreous hemorrhage, and the patient was referred to a tertiary ophthalmology clinic for further evaluation and management.

On examination, best corrected visual acuity (BCVA) was 10/10 (20/20; logMAR 0.0) in the right eye and hand motion in the left eye. Intraocular pressure (IOP) measured 15 mmHg and 14 mmHg, respectively. Slit-lamp biomicroscopy of the left eye revealed dense vitreous hemorrhage (grade 4) without anterior chamber cells or tobacco dust sign. B-scan ultrasonography was not performed, as it was not immediately available at the time of presentation. The right eye was normal.

The patient’s ocular history included amblyopia and congenital cataract in the left eye, as well as glaucoma diagnosed approximately 10 years earlier, managed with topical antiglaucoma medications in both eyes. He had undergone cataract extraction in the left eye 10 years prior, complicated by postoperative IOP elevation, which was subsequently controlled medically. There was no history of ocular trauma, previous vitreous hemorrhage, or retinal detachment. His medical history was notable for hypertension, hypercholesterolemia, and hypothyroidism, all under regular treatment.

**Figure 1:**
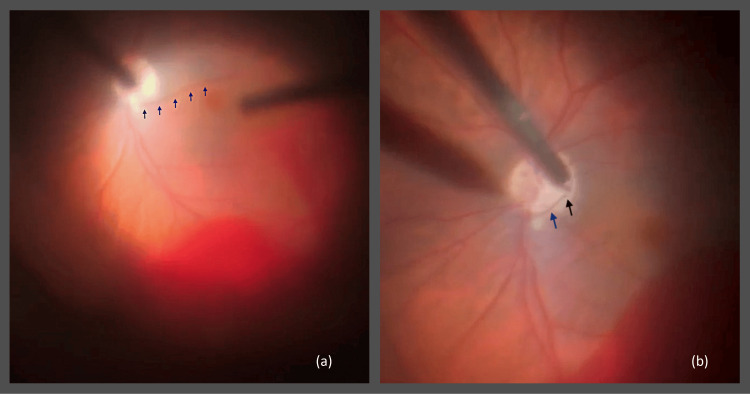
Intraoperative findings during pars plana vitrectomy. (a) Actively bleeding persistent hyaloid artery (blue arrows). (b) Persistent hyaloid artery (blue arrow) and the application point of endocautery (black arrow).

A three-port 25-gauge pars plana vitrectomy was performed within 24 hours of presentation. Intraoperatively, extensive vitreous hemorrhage was confirmed. Following clearance of the hemorrhagic vitreous, an actively bleeding PHA was identified (Figure 1a). No retinal tears, retinal detachment, or neovascularization were observed. Endocautery was applied to the vessel (Figure 1b), followed by fluid-air exchange. All sclerotomies were sutured with 8-0 Vicryl, and subconjunctival cefuroxime and dexamethasone were administered. The procedure lasted approximately 45 minutes and was uneventful (Video 1).

**Video 1:** Real-time intraoperative documentation of an actively bleeding persistent hyaloid artery during pars plana vitrectomy, demonstrating identification of the bleeding vessel and its coagulation with endocautery.

**Figure 2:**
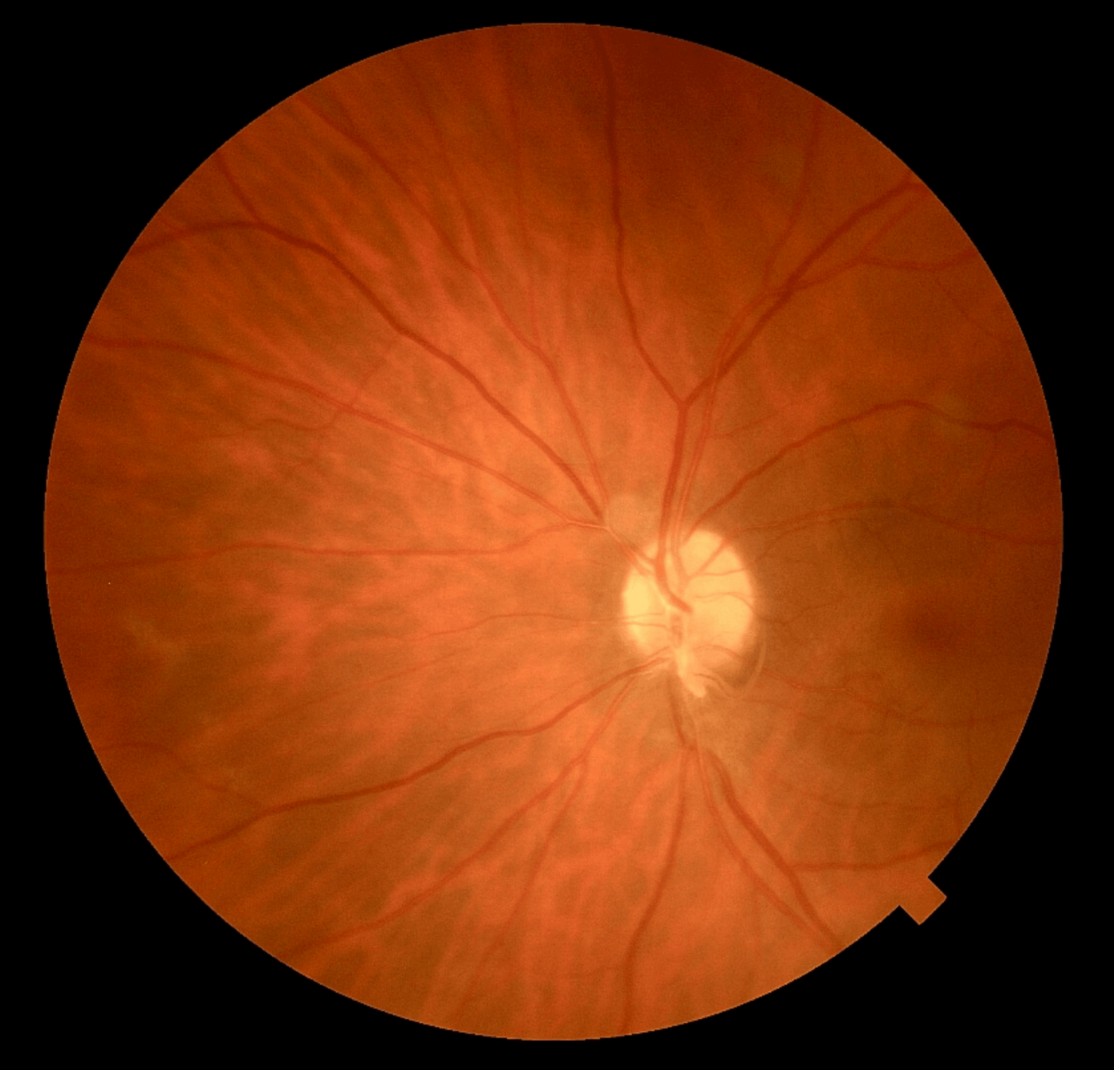
Postoperative color fundus photograph of the left eye (two weeks after surgery), showing a normal fundus without hemorrhage, traction, or retinal detachment.

Postoperatively, the patient was managed with a standard protocol consisting of topical steroid and antibiotic drops for four weeks, demonstrating progressive visual recovery. Two weeks after surgery, BCVA improved to 5/10 (20/40; logMAR 0.3) in the left eye, corresponding to his baseline visual acuity due to amblyopia. IOP remained within normal limits (14 mmHg in both eyes). Postoperative color fundus photography confirmed normal fundus anatomy without hemorrhage, traction, or retinal detachment (Figure 2). The right eye remained normal throughout follow-up.

## Discussion

PFV encompasses a spectrum of abnormalities resulting from the failure of the embryonic vascular system to regress, leading to a wide range of clinical phenotypes [8]. Hyaloid artery remnants are common in neonates, and while the majority spontaneously obliterate in the early postnatal period, persistent residual cases are frequently discovered as incidental findings during routine ophthalmic examination. In other instances, these remnants are associated with secondary pathologies, and in exceedingly rare cases, a persistent vessel may rupture, leading to acute vitreous hemorrhage and sudden vision loss, creating a significant diagnostic challenge [6].

The differential diagnosis of acute vitreous hemorrhage is broad, including several vitreoretinal, retinovascular, choroidal, traumatic, postoperative, neoplastic, inflammatory, and systemic conditions [5]. Because PFV-related hemorrhage is almost exclusively reported in young individuals, PHA rupture is rarely considered in older patients and is often neglected in routine practice. A review of the literature reveals that symptomatic PHA rupture is exceptional beyond early adulthood, with only one case describing a 52-year-old woman who presented with PHA-related vitreous hemorrhage likely triggered by posterior vitreous detachment [9]. The presentation in our 70-year-old patient is therefore highly unusual and broadens the known age spectrum of PHA-related complications. This case further underscores the importance of including congenital anomalies, alongside other etiologies, in the differential diagnosis of spontaneous vitreous hemorrhage in elderly patients, despite age-based expectations.

Pars plana vitrectomy remains the standard of care for non-clearing vitreous hemorrhage [6]. Early surgical intervention is often essential. This is particularly indicated in cases of dense, non-clearing vitreous hemorrhage, inability to visualize the posterior pole, suspected retinal tears or detachment, or when the etiology of hemorrhage requires direct intraoperative assessment. Early vitrectomy permits direct visualization of the bleeding source, establishes a definitive diagnosis, and enables effective removal of hemorrhagic vitreous. In our case, surgery allowed the identification and cauterization of the actively bleeding PHA, preventing recurrent hemorrhage and promoting visual recovery. Endocautery was used to control the hemorrhage, as it provides immediate and precise hemostasis for actively bleeding hyaloid remnants, which are free-floating within the vitreous and do not respond adequately to laser energy.

Interestingly, no hyaloid remnant had been documented in previous funduscopic examinations, even after cataract extraction. A plausible explanation is that highly involuted or fibrotic remnants of the hyaloid artery can be extremely subtle, appearing as fine, translucent strands that are easily overlooked during routine dilated fundus examination. In pseudophakic eyes, visualization of such delicate vitreous structures may be further limited, as these remnants may mimic benign vitreous condensations. Importantly, even when an embryonic vessel appears anatomically insignificant, it may retain microscopic patency for decades, predisposing it to sudden rupture. This interpretation is further supported by the absence of documented cases of hyaloid artery recanalization in the literature.

Although ocular ultrasonography may have provided additional diagnostic information [10], it was not immediately available, and the density of the hemorrhage, combined with the small size of the vessel - may have hindered imaging. As in the case reported by Yu et al., where B-scan ultrasonography failed to visualize a persistent vessel in a 68-year-old patient with incidental PFV despite the absence of vitreous opacities [8], ultrasonography in our patient may also have been of limited diagnostic value.

Το the best of our knowledge, this is the first report in which an actively bleeding PHA has been visualized live during surgery and documented on video, providing unique, direct evidence of this rare mechanism of vitreous hemorrhage.

## Conclusions

This case highlights a rare cause of vitreous hemorrhage in an elderly patient due to rupture of a PHA, a condition typically associated with early life. Although uncommon, PHA should remain in the differential diagnosis of spontaneous vitreous hemorrhage, particularly in patients without systemic or retinal vascular disease. Clinicians should consider PHA particularly when more common causes of vitreous hemorrhage have been excluded. Early surgical intervention allows both definitive diagnosis and effective management, leading to favorable visual and anatomical outcomes.
